# Prevalence and Incidence of Traumatic Experiences Among Orphans in Institutional and Family-Based Settings in 5 Low- and Middle-Income Countries: A Longitudinal Study

**DOI:** 10.9745/GHSP-D-15-00093

**Published:** 2015-08-25

**Authors:** Christine L Gray, Brian W Pence, Jan Ostermann, Rachel A Whetten, Karen O’Donnell, Nathan M Thielman, Kathryn Whetten

**Affiliations:** ^a^​ University of North Carolina at Chapel Hill, Gillings School of Global Public Health, Department of Epidemiology, Chapel Hill, NC, USA; ^b^​ Duke University, Duke Global Health Institute, Center for Health Policy and Inequalities Research, Durham, NC, USA; ^c^​ Duke University, Center for Child and Family Health, Durham, NC, USA; ^d^​ Duke University, Division of Infectious Diseases and International Health, Department of Medicine, Durham, NC, USA; ^e^​ Duke University, Terry Sanford Institute of Public Policy, Durham, NC, USA

## Abstract

**Background:**

Policy makers struggling to protect the 153 million orphaned and separated children (OSC) worldwide need evidence-based research on the burden of potentially traumatic events (PTEs) and the relative risk of PTEs across different types of care settings.

**Methods:**

The Positive Outcomes for Orphans study used a 2-stage, cluster-randomized sampling design to identify 1,357 institution-dwelling and 1,480 family-dwelling orphaned and separated children in 5 low- and middle-income countries (LMICs) in sub-Saharan Africa and Asia. We used the Life Events Checklist developed by the National Center for Posttraumatic Stress Disorder to examine self-reported PTEs among 2,235 OSC ages 10–13 at baseline. We estimated prevalence and incidence during 36-months of follow-up and compared the risk of PTEs across care settings. Data collection began between May 2006 and February 2008, depending on the site.

**Results:**

Lifetime prevalence by age 13 of any PTE, excluding loss of a parent, was 91.0% (95% confidence interval (CI) = 85.6, 94.5) in institution-dwelling OSC and 92.4% (95% CI = 90.3, 94.0) in family-dwelling OSC; annual incidence of any PTE was lower in institution-dwelling (23.6% [95% CI = 19.4, 28.7]) than family-dwelling OSC (30.0% [95% CI = 28.1, 32.2]). More than half of children in institutions (50.3% [95% CI = 42.5, 58.0]) and in family-based care (54.0% [95% CI = 50.2, 57.7]) had experienced physical or sexual abuse by age 13. Annual incidence of physical or sexual abuse was lower in institution-dwelling (12.9% [95% CI = 9.6, 17.3]) than family-dwelling OSC (19.4% [95% CI = 17.7, 21.3]), indicating statistically lower risk in institution-dwelling OSC (risk difference = 6.5% [95% CI = 1.4, 11.7]).

**Conclusion:**

Prevalence and incidence of PTEs were high among OSC, but contrary to common assumptions, OSC living in institutions did not report more PTEs or more abuse than OSC living with families. Current efforts to reduce the number of institution-dwelling OSC may not reduce incidence of PTEs in this vulnerable population. Protection of children from PTEs should be a primary consideration, regardless of the care setting.

## Abstract (precis)

Contrary to some conventional wisdom, in this large study that randomly sampled orphans and separated children from 5 countries, prevalence of reported traumatic events was no worse among those institutionalized than among those in family-based care. Reported incidence of physical or sexual abuse was actually higher for those in family-based care. Understanding the specific context, and elements contributing to potential harm and benefits in both family-based and institutional care, are essential to promoting the best interest of the child.

## INTRODUCTION

The appropriateness of institutional care for orphaned children has become a central question for international aid policy affecting many low-and middle-income countries (LMICs). Research has convincingly demonstrated that infants raised in emotionally and socially deprived orphanages in Eastern Europe suffered significant cognitive delays and long-term negative effects.^1^^-^^4^ A meta-analysis of 42 studies in 19 countries also reported lower Intelligence Quotient (IQ) values among institutionalized children compared with those in family-based care.^5^

In the meta-analysis, however, differences between institutionalized and non-institutionalized children were not observed in 3 of the 4 countries ranking low on the Human Development Index, a widely accepted measure of health, education, and standard of living, suggesting that conclusions about what is broadly labeled “institution” may not hold for institutionalized orphans in LMICs. Yet the findings from the literature have led to a generalization that all institutional care is harmful to all children and have fueled international policies aimed at reducing the number of institutions and using them exclusively as a temporary option until family-based care can be reestablished.^6^^-^^8^ At the same time, the numbers of street children are on the rise in these same countries. Furthermore, quality of care in institutional settings varies substantially, and more recent research in a wide range of countries has suggested that orphans raised in institutions have comparable health and cognitive outcomes as their peers raised in family-based settings.^9^^-^^11^

Among the concerns raised about institutional care for orphans is the perceived increased risk for traumatic experiences, such as sexual abuse and physical assault. In addition to the suffering imposed by the trauma itself, traumatic experiences can have long-lasting consequences for children, including adverse effects on their achievements and functioning, even if the children do not meet criteria for posttraumatic stress disorder.^12^^-^^15^ While several studies have described abuse, neglect, and adversity of orphans in institutional settings,^16^^-^^20^ these studies were limited by small sample sizes and were not designed to compare experiences of orphans in different settings. Furthermore, a systematic review of 15 studies suggested that orphans living in extended families in sub-Saharan Africa suffered from substantial maltreatment.^21^ Importantly, no longitudinal studies compare institutions with family-based settings based on statistically representative samples of orphans to estimate prevalence and incidence of exposure to traumatic events.

Orphans in institutional settings are perceived to be at higher risk for traumatic experiences than those raised in family settings.

The purpose of this study is to estimate the lifetime prevalence and annual incidence of potentially traumatic events experienced by statistically representative samples of orphaned or separated children (OSC) living in institutional and family-based settings in 5 culturally diverse LMICs. Specifically, we examine the hypothesis that orphans in institutional care experience more potentially traumatic events than their counterparts living in family-based settings.

## METHODS

### Population

We used data from the Positive Outcomes for Orphans (POFO) study, a longitudinal study conducted at 6 culturally, politically, and geographically diverse sites in 5 LMICs: Battambang District, Cambodia; Addis Ababa, Ethiopia; Hyderabad, India; Nagaland, India; Bungoma District, Kenya; and Kilimanjaro Region, Tanzania.

A 2-stage sampling design was used to identify a random sample of 1,480 family-dwelling OSC and a random sample of 1,357 institution-dwelling OSC ages 6–12 years old at baseline. Data collection began between May 2006 and February 2008, depending on the site. The samples were designed to be statistically representative of the institution-dwelling and family-dwelling OSC populations in the regions from which children were selected.

### Sampling Frame

#### Institution-Dwelling Children

An institution was defined as a structure with at least 5 OSC from at least 2 different families with caregivers not biologically related to the OSC. The number of children per institution ranged from 5 to 376, with a mean of 63 and median of 42 children; approximately 35% of institutions in the study had 25 children or fewer.^22^ Comprehensive lists of all institutions in each of the 6 sites were developed through inquiries with local government officials, schools, and organizations working with orphans. For each site, institutions were randomized for selection and sequentially approached until 250 total children were enrolled. Up to 20 children ages 6–12 were randomly selected from each institution; if there were fewer than 20 age-eligible children in the institution, all were selected. At 3 sites, the number of children selected per institution was increased to attain the target sample of 250 per site.

#### Family-Dwelling Children

Fifty sampling areas (“clusters”) were defined for each study site using geographic or administrative boundaries. Up to 5 children from each cluster were selected through random sampling of available lists or through a house-to-house census. Some clusters had 6 to 10 children enrolled due to substitutions from clusters with insufficient enrollment or insufficient numbers of eligible children. In homes with more than 1 age-eligible child, the child whose first name was first alphabetically was selected. Most, but not all, family-dwelling OSC lived with the remaining parent or other biological relatives.

### Measures

#### Administration of Questionnaire

Children were interviewed at approximately 6-month intervals for up to 3 years for a total of 7 rounds of data collection. Trauma measures were administered at baseline and annually during follow-up, for up to 4 assessments for each child included in the analysis.

#### Potentially Traumatic Events

We used potentially traumatic experiences self-reported by OSC ages ≥10 at the time of assessment; based on both pilot testing as well as Institutional Review Board (IRB) recommendations, children younger than 10 were not administered trauma assessments. This analysis uses self-reported trauma exposures because a prior study in this population showed discordance between self-report and caregiver report, with caregivers reporting significantly fewer potentially traumatic events.^23^ This discordance is consistent with underreporting of child trauma exposure by parents and caregivers in other populations.^24^^-^^26^ In most cases, caregivers of orphans have not been present for the orphan’s entire childhood and may have limited knowledge of the child’s trauma history and are also less likely to report violence and abuse within the current caregiving setting.

Potentially traumatic experiences were assessed at baseline and at 3 annual follow-up surveys, using the Life Events Checklist (LEC) developed by the National Center for Posttraumatic Stress Disorder (PTSD) to aid in PTSD detection.^27^ The LEC has been widely used in cross-cultural settings and is predictive of anxiety, depression, and PTSD.^28^ Children responded to a list of 17 “things I have seen and heard,” indicating whether the event had been experienced 1 time, more than 1 time, or not at all; at follow-up assessments the child also indicated if he or she had experienced the event in the past year (i.e., approximately since the last trauma assessment), prior to the past year, or both.

For analysis, the 17 PTEs were collapsed into 6 categories: disasters or accidents; war, riots, or killings; physical or sexual abuse; witnessing violence in the care setting; witnessing family death; and being forced to leave the care setting (Supplementary Table A). These categories are the same as those used previously in similar work, as well as to describe PTEs in this population.^9^^,^^29^ Being an orphan in and of itself was not included as a traumatic event because that was a defining characteristic for inclusion in the study. However, if the child personally watched the death of their parent happen, that was included in the “witnessing family death” category.

Lifetime prevalence was assessed at baseline and at each annual follow-up interview. At follow-up interviews, children were also asked whether each type of event had been experienced in the past year. In this analysis, incident trauma was defined as reporting having experienced the event within the past year, regardless of whether the child had experienced the event previously.

#### Child Characteristics

Demographic information such as gender, age, setting (institution-dwelling or family-dwelling), OSC type (single orphan, double orphan, or separated; maternal death, paternal death, or both) were collected at baseline.

### Analyses

Because trauma measures were not administered to children under age 10, age-specific estimates of lifetime prevalence and 12-month incidence of PTEs were calculated based on interviews at which the child’s current age was at least 10 years old.

We used logistic regression to estimate the lifetime prevalence of trauma reported by age 13 in each of the 6 trauma categories, as well as lifetime prevalence of any trauma. We used current age, a squared term for current age, and study round in the models and calculated lifetime predicted prevalence at age 13 in round 7 (the final round of follow-up). We predicted lifetime prevalence at age 13 because most study participants included in this analysis were age 13 at some time during follow-up.

We estimated the proportion and 95% confidence interval (CI) of participants reporting any PTE and each type of PTE in the past year as the 12-month incidence using log binomial regression.

To provide a direct estimate comparing the prevalence difference for each trauma category between OSC in institution-based care and those in family-based care, we used a linear risk model (identity link and binomial distribution) that included a parameter estimate for the setting (family-based vs. institution-based), age centered at 13, a squared term for age centered at 13, and product terms between setting and each of the age terms. The parameter estimate for setting is reported as the prevalence difference (PD) and 95% CI.

To provide a direct comparison of incidence, we used a linear risk model with a term for setting (family-based vs. institution-based); that parameter estimate is reported as a risk difference (RD) and 95% CI.

Finally, between institutional and family-based care, we compared cumulative prevalence and annual incidence by year of age (10–15 years) for any PTE reported and for physical or sexual abuse specifically. The few 16-year-old participants at the final round of data collection were combined with 15-year-olds to prevent unstable estimates.

All prevalence and incidence estimates and CIs described above accounted for the complex survey design through incorporation of sampling weights and specification of the site and sampling unit levels of the design, as previously described.^22^ All analyses were conducted using Stata 13.^30^

### Ethical Approval

The POFO study was approved by the IRB at Duke University and by the IRB at each of the study sites. Caregiver consent and child assent were obtained and recorded on IRB-approved consent forms. Local interviewers were trained on site-specific protocols created for this study for addressing reported or observed abuse of children. This included an advisory board consisting of local child professionals to which reports of abuse and other difficult situations were reported. All study personnel were trained in maintaining confidentiality of all information shared in the course of data collection or analysis. All data are kept on a secure server accessible only by study personnel who had completed IRB training, including local study site coordinators. For follow-up, key personnel accessed the minimal information necessary to locate a child.

## RESULTS

### Sample Characteristics

In total, 2,235 OSC (1,182 family-dwelling and 1,053 institution-dwelling) were ≥10 years old at 1 or more interviews and were included in this analysis (Table 1). Over half of OSC (58% in institutions and 53% in family-based care) in this analysis were male, and most (94% in institutions and 96% in family-based care) had their first trauma interview by or at age 12. A greater percentage of institution-dwelling (39%) than family-dwelling OSC (18%) were double orphans. Among single orphans, 77% of institution-dwelling OSC and 78% of family-dwelling OSC had lost their father.

**TABLE 1:** Characteristics of OSC in Institution-Based and Family-Based Care (N = 2,235)

Characteristic	No. (%) of Institution-Dwelling OSC (n = 1,053)	No. (%) of Family-Dwelling OSC (n = 1,182)
Sex		
Male	614 (58.3)	631 (53.4)
Female	439 (41.7)	551 (46.6)
Site		
Cambodia	112 (10.6)	199 (16.8)
Ethiopia	175 (16.6)	192 (16.2)
Hyderabad (India)	209 (19.8)	222 (18.8)
Kenya	188 (17.9)	192 (16.2)
Nagaland (India)	150 (14.2)	163 (13.8)
Tanzania	219 (20.8)	214 (18.1)
Age at first trauma interview, years		
10	395 (37.5)	457 (38.7)
11	379 (36.0)	462 (39.1)
12	214 (20.3)	210 (17.8)
13	56 (5.3)	45 (3.8)
14	4 (0.4)	7 (0.6)
15	5 (0.5)	1 (0.1)
OSC deceased parent		
Neither (separated)	178 (16.9)	120 (10.2)
Mother	105 (10.0)	188 (15.9)
Father	358 (34.0)	663 (56.1)
Both	412 (39.1)	211 (17.9)

Abbreviation: OSC, orphaned or separated children.

### Prevalence and Incidence of Overall Trauma

By age 13, over 90% of children both in institutions and in family-based care had experienced at least 1 PTE beyond the loss of a parent; the predicted lifetime prevalence of any trauma was approximately the same in both settings: 91.0% (95% CI = 85.6, 94.5) in institution-based care vs. 92.4% (95% CI = 90.3, 94.0) in family-based care (Table 2). Annual incidence of any trauma was lower in institution-dwelling OSC (23.6% [95% CI = 19.4, 28.7]) than in family-dwelling OSC (30.0% [95% CI = 28.1, 32.2]), but the risk difference was not statistically significant (RD = 6.4% [95% CI = -0.2, 13.0]) (Table 3).

By age 13, nearly all children had experienced at least 1 potentially traumatic event beyond the loss of a parent.

**TABLE 2:** Prevalence of Lifetime Trauma by Age 13 Among Orphaned or Separated Children (OSC) by Care Setting, 6 Sites in 5 Low- and Middle-Income Countries^a^

	Institution-Based OSC		Family-Based OSC		Prevalence Difference^b^	
	%	95% CI	%	95% CI	%	95% CI
Any trauma	91.0	(85.6, 94.5)	92.4	(90.3, 94.0)	0.0	(−4.4, 4.4)
Witnessing a family death	72.5	(67.6, 76.9)	71.8	(68.5, 74.9)	−2.7	(−9.0, 3.5)
Physical or sexual abuse	50.3	(42.5, 58.0)	54.0	(50.2, 57.7)	3.5	(−7.9, 15.0)
Violence in family or care setting	30.9	(25.1, 37.4)	36.8	(33.1, 40.7)	6.6	(−3.6, 16.8)
Forced to leave home or care setting	27.6	(21.2, 35.2)	15.0	(12.5, 17.9)	−14.8	(−24.8, −4.8)
War, riots, killings	20.0	(14.6, 26.8)	22.0	(19.2, 25.0)	−1.6	(−9.1, 5.9)
Disaster or accidents	5.9	(3.7, 9.5)	9.5	(7.5, 11.9)	3.5	(−1.7, 8.8)

^a^​ Battambang District, Cambodia; Addis Ababa, Ethiopia; Hyderabad, India; Nagaland, India; Bungoma District, Kenya; and Kilimanjaro Region, Tanzania.

^b^​ Differences were modeled separately from the prevalence estimates and may be slightly different than an exact subtraction of the institution-based and family-based prevalences. Institution-based OSC were the referent; positive differences indicate family-based OSC had higher prevalence while negative differences indicate institution-based OSC had higher prevalence.

**TABLE 3:** Annual Trauma Incidence Among Orphaned and Separated Children (OSC) Over the Course of the Study,^a^ by Care Setting, 6 Sites in 5 Low- and Middle-Income Countries^b^

	Institution-Based OSC		Family-Based OSC		Risk Difference^c^	
	%	95% CI	%	95% CI	%	95% CI
Any trauma	23.6	(19.4, 28.7)	30.0	(28.1, 32.2)	6.4	(−0.2, 13.0)
Witnessing a family death	3.6	(2.5, 5.2)	6.0	(5.1, 7.0)	2.3	(0.5, 4.2)
Physical or sexual abuse	12.9	(9.6, 17.3)	19.4	(17.7, 21.3)	6.5	(1.4, 11.7)
Violence in family or care setting	6.6	(4.9, 9.0)	9.1	(7.7, 10.6)	2.4	(−0.5, 5.4)
Forced to leave home or care setting	3.4	(1.9, 6.1)	1.0	(0.7, 1.6)	−2.4	(−4.6, −0.1)
War, riots, killings	5.1	(3.4, 7.5)	5.8	(4.9, 7.0)	0.8	(−2.4, 3.9)
Disaster or accidents	0.7	(0.3, 1.5)	0.9	(0.6, 1.4)	0.2	(−0.5, 0.9)

^a^​ Data collection began between May 2006 and February 2008, depending on the site, and continued for 36 months of follow-up.

^b^​ Battambang District, Cambodia; Addis Ababa, Ethiopia; Hyderabad, India; Nagaland, India; Bungoma District, Kenya; and Kilimanjaro Region, Tanzania.

^c^​ Differences were modeled separately from the incidence estimates and may be slightly different than an exact subtraction of the incidence-based and family-based incidences. Institution-based OSC were the referent; positive differences indicate family-based OSC had higher risk while negative differences indicate institution-based OSC had higher risk.

Follow-up subgroup analyses (not shown) indicated similar results. We observed no gender differences in prevalence or incidence of any trauma in either setting: 91.7% (95% CI = 85.0, 95.5) of males and 90.3% (95% CI = 84.2, 94.1) of females in institution-based care compared with 92.0% (95% CI = 89.0, 94.2) of males and 92.9% (95% CI = 89.8, 95.1) of females in family-based care reported any trauma. While there was some variation in both prevalence and incidence of any trauma by study site, there were no differences between institution-dwelling and family-dwelling OSC at any site.

### Prevalence and Incidence of Specific Types of Trauma

The most commonly experienced category of trauma was witnessing a family death, reported by 72.5% (95% CI = 67.6, 76.9) of institution-dwelling and 71.8% (95% CI = 68.5, 74.9) of family-dwelling OSC (Table 2). Endorsement of this category means the child personally saw the death of a family member, whether from illness or violence, including watching the death of a parent.

More than half of children had experienced physical or sexual abuse by age 13.

More than half of children both in institutions (50.3% [95% CI = 42.5, 58.0]) and in family-based care (54.0% [95% CI = 50.2, 57.7] had experienced physical or sexual abuse by age 13 (Table 2). In both settings, the 12-month incidence of physical or sexual abuse had the highest incidence (>13%) relative to other trauma categories, which all had an incidence of less than 10% (Table 3). Institution-dwelling OSC had similar or lower predicted prevalence of PTEs than family-dwelling OSC for each type of trauma except being forced to leave the care setting (Table 2). Similarly, annual incidence of PTEs was similar or lower among institution-dwelling OSC for all trauma types except being forced to leave home (Table 3). In particular, the annual incidence of physical or sexual abuse was 12.9% (95% CI = 9.6, 17.3) in institution-based care, compared with 19.4% (95% CI = 17.7, 21.3) in family-based care, indicating statistically significantly higher risk in family-dwelling OSC (RD = 6.5% [95% CI = 1.4, 11.7]) (Table 3).

Annual incidence of physical or sexual abuse was significantly higher among orphans in family-based care (19%) than in institutional care (13%).

The Figure compares cumulative prevalence and annual incidence of any trauma and of physical or sexual abuse at each age (10–15 years) across settings. In general, estimates at each age are similar but slightly higher in family-based settings than in institution-based settings.

**FIGURE:**
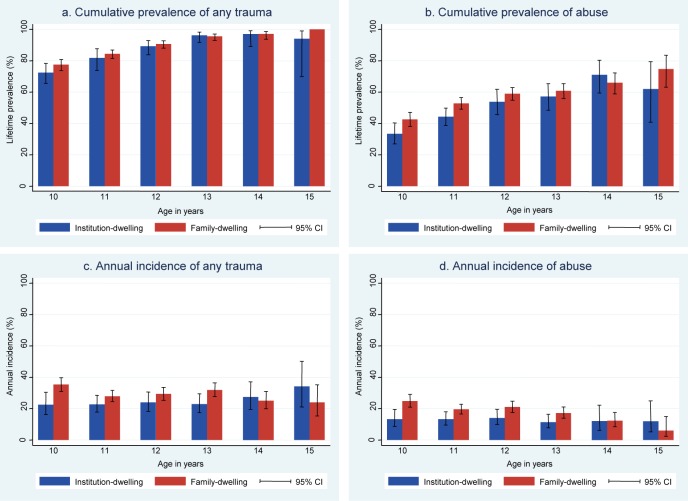
Prevalence and Incidence of Any Trauma and of Abuse by Age in Institution-Based vs. Family-Based Settings

## DISCUSSION

This study of a large and statistically representative sample of orphans and separated children from 5 LMICs, followed prospectively for 3 years, documented a substantial burden of potentially traumatic events that extended beyond the trauma of losing a parent. Nearly all OSC—regardless of care setting—had experienced at least 1 PTE by age 13, nearly three-quarters had witnessed a family death, and half had experienced physical or sexual abuse. Moreover, exposure to PTEs continued throughout the 3-year follow-up period of the study, during which time 24% of institution-dwelling OSC and 30% of family-dwelling OSC experienced an incident PTE each year, and 13% of institution-dwelling OSC and 19% of family-dwelling OSC experienced physical or sexual abuse each year.

Importantly, we found that over 3 years of longitudinal follow-up, the incidence of PTEs in general, and of physical or sexual abuse in particular, was not higher among institution-dwelling OSC compared with family-dwelling OSC. This finding is noteworthy because deinstitutionalization has been advocated, in part, based on assumptions about pervasive abuse and neglect in institutional care settings relative to other settings.^6^^-^^8^ However, our longitudinal data, designed to draw comparisons across such settings, do not support the conclusion that orphans in institutional care are exposed to potentially traumatic events with greater frequency than their counterparts in family-based care.

Our findings do not support the conclusion that orphans in institutional care are exposed more than those in family-based care to potentially traumatic events.

Physical or sexual abuse had, by far, the highest incidence of any trauma type. Annual risk of physical or sexual abuse was statistically significantly higher for OSC in family-based care relative to those in institution-based care, although estimates were somewhat imprecise. The prevalence of these experiences was comparable in both institutional and family-based settings Because the prevalence rates take into account the life history of the child, it is possible that the similar rates between institutional and family-based children are due to abuse that occurred to institutional-based children before their entry into the institution and may have been the reason for their entry. Another large longitudinal study in Kenya following more than 3,000 children found that of those in institutions, approximately half (52%) had been abused before entering the institution.^31^

Additionally, incidence of abuse at younger ages is higher than incidence at older ages, suggesting that focusing on identifying trauma, particularly abuse, in younger children may increase the opportunity for intervention and provision of support services. Younger children are perhaps at greater risk for abuse from other children and adults due to their smaller physical size and the ability to manipulate them emotionally. The higher prevalence and incidence of being forced to leave the home or care setting among institution-dwelling than family-based OSC may be attributable, in part, to closing of institutions. Interventions targeting communities with OSC should focus on preventing abuse and providing counseling and support for survivors of abuse, in both types of settings.

Incidence of abuse is higher at younger than older ages.

Poverty underlies the conditions in which many OSC live; protection from abuse can be difficult if caregivers spend long hours working outside the home or if the perpetrator is a family member. Community-wide efforts may provide preventive measures and protection. Emerging interventions tailored to resource-poor settings may mitigate sequelae from PTEs; at least 1 recent trauma-focused cognitive behavioral therapy program has shown promise and can be provided by trained lay professionals.^32^^,^^33^

Two key characteristics differentiate this work from previous research that demonstrated significant and long-term impairment in institutional-based orphans in Eastern Europe.^1^^-^^4^ First, prior studies focused on particularly problematic institutions where gross negligence was known to have occurred. In contrast, the present study used a probabilistic sampling method based on a census of institutions in a defined geographic area to recruit a cohort of OSC statistically representative of the population of orphans and separated children living in institutional care. Second, the prior research focused on orphans who had entered the institution in infancy, whereas the present study recruited children ages 6–12, of whom only a small portion had entered institutional care in infancy. Given that 95% of children who are orphaned and in need of care are over the age of 5, it is critically important that policies for these children be based on evidence derived from those over the age of 5.

### Strengths and Limitations

This study has several important strengths. The POFO data were collected longitudinally for 3 years on representative samples of both institution-dwelling and family-dwelling OSC using a cluster-randomized design, with a very high rate of 3-year follow-up (82%). Retention in this analytic sample is difficult to summarize since children enter at later rounds as they become eligible at age 10 to answer trauma-related questions, but Supplementary Table B demonstrates the overall POFO retention. A prior analysis showed that follow-up did not differ by care setting.^34^ The 6 study sites in 5 LMICs reflect broad cultural, geographic, and economic diversity. Importantly, this study incorporates institution-dwelling OSC and longitudinal follow-up to expand upon earlier studies in the POFO population. To our knowledge, no other study uses longitudinal data to quantify the lifetime prevalence or annual incidence of traumatic experiences among OSC in sub-Saharan African and Asian regions, which account for over two-thirds of the world’s OSC population.^35^ Furthermore, we describe the prevalence and incidence among specific categories of trauma and provide evidence against the hypothesis that physical and sexual abuse is more pervasive in institutions than in family-based settings.

We note several limitations to our study. First, reporting bias is a possibility. Traumatic events are likely to be underreported, implying this study underestimates the actual burden of PTEs in this population. We do not have reason to believe that this bias would be stronger in one setting or the other. Second, the total number of traumatic events is unknown; respondents endorsed occurrence of each trauma as “never,” “one time,” or “two or more times.” Third, the chronology of traumatic events with respect to orphaning (which events came before or after being orphaned) is unknown. Fourth, although our study included diverse LMICs, South America and Eastern Europe were not represented. We recognize our results may not be generalizable to those cultural contexts. Finally, although the POFO study has a sample of non-OSC for comparison, it is too small to include in these analyses. Therefore, we did not include those estimates in the present paper.

## CONCLUSION

Caring for the sheer number of OSC worldwide (153 million, including 17 million orphaned by AIDS) presents a complex problem that demands evidence-based solutions.^35^ When developing interventions or policies for this vulnerable population, the magnitude of PTEs is a critical concern. While protecting OSC from trauma in general and abuse in particular must be a high priority, the results presented here suggest that risk of trauma and abuse is not restricted to OSC living in institutional care but is at least equally common among OSC living in family-based settings. Protection of children from PTEs should be a primary consideration in care for OSC, regardless of setting.

Protection of orphans and separated children from potentially traumatic events should be a primary consideration, regardless of the care setting.

## Notes

**Peer Reviewed**

## References

[1] Rutter M Beckett C Castle J Colvert E Kreppner J Mehta M Effects of profound early institutional deprivation: an overview of findings from a UK longitudinal study of Romanian adoptees. Eur J Dev Psychol. 2007;4(3):332–350. 10.1080/17405620701401846

[2] Zeanah CH Egger HL Smyke AT Nelson CA Fox NA Marshall PJ Institutional rearing and psychiatric disorders in Romanian preschool children. Am J Psychiatry. 2009;166(7):777–785. 10.1176/appi.ajp.2009.08091438. 19487394

[3] Zeanah CH Nelson CA Fox NA Smyke AT Marshall P Parker SW Designing research to study the effects of institutionalization on brain and behavioral development: the Bucharest Early Intervention Project. Dev Psychopathol. 2003;15(04):885–907. 10.1017/S0954579403000452. 14984131

[4] Rus AV Stativa E Pennings JS Cross DR Ekas N Purvis KB Severe punishment of children by staff in Romanian placement centers for school-aged children: effects of child and institutional characteristics. Child Abuse Negl. 2013;37(12):1152–1162. 10.1016/j.chiabu.2013.07.003. 23932392

[5] van IJzendoorn MH Juffer F Poelhuis CWK. Adoption and cognitive development: a meta-analytic comparison of adopted and nonadopted children’s IQ and school performance. Psychol Bull. 2005;131(2):301–316. 10.1037/0033-2909.131.2.301. 15740423

[6] United Nations Children’s Fund (UNICEF) Progress for children: a report card on child protection. New York: UNICEF; 2009 Available from: http://www.unicef.org/publications/files/Progress_for_Children-No.8_EN_081309.pdf

[7] Government of the United States (USG) United States Government action plan on children in adversity: a framework for international assistance: 2012–2017. Washington (DC): USG; 2012 Available from: http://www.usaid.gov/sites/default/files/documents/1860/United%20States%20Action%20Plan%20on%20Children%20in%20Adversity.pdf

[8] US Congress Children In Families First (CHIFF) Act. H.R. 4143 (113th Congress, 2013-2015) 2014.

[9] Whetten K Ostermann J Whetten R O’Donnell K Thielman N Positive Outcomes for Orphans Research Team. More than the loss of a parent: potentially traumatic events among orphaned and abandoned children. J Trauma Stress. 2011;24(2):174–182. 10.1002/jts.20625. 21442663 PMC3610328

[10] Li X Barnett D Fang X Lin X Zhao G Zhao J Lifetime incidences of traumatic events and mental health among children affected by HIV/AIDS in rural China. J Clin Child Adolesc Psychol. 2009;38(5):731–744. 10.1080/15374410903103601. 20183657

[11] Atwoli L Ayuku D Hogan J Koech J Vreeman RC Ayaya S Impact of domestic care environment on trauma and posttraumatic stress disorder among orphans in western Kenya. PLoS One. 2014;9(3):e89937. 10.1371/journal.pone.0089937. 24625395 PMC3953071

[12] Glaser D. Child abuse and neglect and the brain--a review. J Child Psychol Psychiatry. 2000;41(1):97–116. 10.1111/1469-7610.00551. 10763678

[13] Scrimin S Moscardino U Capello F Axia G. Attention and memory in school-age children surviving the terrorist attack in Beslan, Russia. J Clin Child Adolesc Psychol. 2009;38(3):402–414. 10.1080/15374410902851689. 19437300

[14] Broberg AG Dyregrov A Lilled L. The Goteborg discotheque fire: posttraumatic stress, and school adjustment as reported by the primary victims 18 months later. J Child Psychol Psychiatry. 2005;46(12):1279–1286. 10.1111/j.1469-7610.2005.01439.x. 16313428

[15] Giaconia RM Reinherz HZ Silverman AB Pakiz B Frost AK Cohen E. Traumas and posttraumatic stress disorder in a community population of older adolescents. J Am Acad Child Adolesc Psychiatry. 1995;34(10):1369–1380. 10.1097/00004583-199510000-00023. 7592275

[16] Boucher S Paré N Perry JC Sigal JJ Ouimet MC. Consequences of an institutionalized childhood: the case of the “Duplessis orphans”. Sante Ment Que. 2008;33(2):271–291. 10.7202/019678ar. 19370267

[17] Perry JC Sigal JJ Boucher S Paré N. Seven institutionalized children and their adaptation in late adulthood: the children of Duplessis (Les Enfants de Duplessis). Psychiatry. 2006;69(4):283–301. 10.1521/psyc.2006.69.4.283. 17326727

[18] Wanat S Whisnant J Reicherter D Solvason B Juul S Penrose B Coping with the challenges of living in an Indonesian residential institution. Health Policy. 2010;96(1):45–50. 10.1016/j.healthpol.2010.01.001. 20102784

[19] McCall RB. The consequences of early institutionalization: can institutions be improved? Should they? Child Adolesc Ment Health. 2013;18(4). 24273458 10.1111/camh.12025 PMC3833822

[20] Karadağ Çaman Ö Özcebe H. Adolescents living in orphanages in Ankara: psychological symptoms, level of physical activity, and associated factors. Turk Psikiyatri Derg. 2011;22(2):93–103. 21638231

[21] Morantz G Cole D Vreeman R Ayaya S Ayuku D Braitstein P. Child abuse and neglect among orphaned children and youth living in extended families in sub-Saharan Africa: what have we learned from qualitative inquiry? Vulnerable Child Youth Stud. 2013;8(4):338–352. 10.1080/17450128.2013.764476. 24563656 PMC3929282

[22] Whetten K Ostermann J Whetten RA Pence BW O’Donnell K Messer LC Positive Outcomes for Orphans (POFO) Research Team. A comparison of the wellbeing of orphans and abandoned children ages 6–12 in institutional and community-based care settings in 5 less wealthy nations. PLoS One. 2009;4(12):e8169. 10.1371/journal.pone.0008169. 20020037 PMC2790618

[23] Guru Rajan D Shirey K Ostermann J Whetten R O’Donnell K Whetten K. Child and caregiver concordance of potentially traumatic events experienced by orphaned and abandoned children. Vulnerable Child Youth Stud. 2014;9(3):220–233. 10.1080/17450128.2013.855346. 25379051 PMC4217223

[24] Lewis T Thompson R Kotch JB Proctor LJ Litrownik AJ English DJ Parent-youth discordance about youth-witnessed violence: associations with trauma symptoms and service use in an at-risk sample. Child Abuse Negl. 2012;36(11-12):790–797. 10.1016/j.chiabu.2012.09.009. 23153569 PMC3762220

[25] Oransky M Hahn H Stover CS. Caregiver and youth agreement regarding youths’ trauma histories: implications for youths’ functioning after exposure to trauma. J Youth Adolesc. 2013;42(10):1528–1542. 10.1007/s10964-013-9947-z. 23580028

[26] Ceballo R Dahl TA Aretakis MT Ramirez C Inner-city children's exposure to community violence: how much do parents know? J Marriage Fam. 2001;63(4):927–940. 10.1111/j.1741-3737.2001.00927.x

[27] Gray MJ Litz BT Hsu JL Lombardo TW. Psychometric properties of the life events checklist. Assessment. 2004;11(4):330–341. 10.1177/1073191104269954. 15486169

[28] Elhai JD Gray MJ Kashdan TB Franklin CL. Which instruments are most commonly used to assess traumatic event exposure and posttraumatic effects? A survey of traumatic stress professionals. J Trauma Stress. 2005;18(5):541–545. 10.1002/jts.20062. 16281252

[29] Mugavero M Ostermann J Whetten K Leserman J Swartz M Stangl D Barriers to antiretroviral adherence: the importance of depression, abuse, and other traumatic events. AIDS Patient Care STDS. 2006;20(6):418–428. 10.1089/apc.2006.20.418. 16789855

[30] StataCorp Stata statistical software: release 13. College Station (TX): StataCorp LP; 2013.

[31] Morantz G Cole DC Ayaya S Ayuku D Braitstein P. Maltreatment experiences and associated factors prior to admission to residential care: a sample of institutionalized children and youth in western Kenya. Child Abuse Negl. 2013;37(10):778–787. 10.1016/j.chiabu.2012.10.007. 23290620 PMC3633719

[32] O’Donnell K Dorsey S Gong W Ostermann J Whetten R Cohen JA Treating maladaptive grief and posttraumatic stress symptoms in orphaned children in Tanzania: group-based trauma-focused cognitive-behavioral therapy. J Trauma Stress. 2014;27(6):664–671. 10.1002/jts.21970. 25418514 PMC4591025

[33] Murray LK Skavenski S Kane JC Mayeya J Dorsey S Cohen JA Effectiveness of trauma-focused cognitive behavioral therapy among trauma-affected children in Lusaka, Zambia: a randomized clinical trial. JAMA Pediatr. Epub 2015 Jun 29. 10.1001/jamapediatrics.2015.0580. 26111066 PMC9067900

[34] Whetten K Ostermann J Pence BW Whetten RA Messer LC Ariely S Positive Outcomes for Orphans (POFO) Research Team. Three-year change in the wellbeing of orphaned and separated children in institutional and family-based care settings in five low- and middle-income countries. PLoS One. 2014;9(8):e104872. 10.1371/journal.pone.0104872. 25162410 PMC4146542

[35] United Nations Children’s Fund (UNICEF) The state of the world's children 2012: children in the urban world. New York: UNICEF; 2012 Available from: http://www.unicef.org/sowc2012/pdfs/SOWC%202012-Main%20Report_EN_13Mar2012.pdf

